# Can Operant Conditioning of EMG-Evoked Responses Help to Target Corticospinal Plasticity for Improving Motor Function in People With Multiple Sclerosis?

**DOI:** 10.3389/fneur.2020.00552

**Published:** 2020-07-15

**Authors:** Aiko K. Thompson, Thomas Sinkjær

**Affiliations:** ^1^Department of Health Sciences and Research, College of Health Professions, Medical University of South Carolina, Charleston, SC, United States; ^2^Department of Health Science and Technology, Aalborg University, Aalborg, Denmark; ^3^Lundbeck Foundation, Copenhagen, Denmark

**Keywords:** operant conditioning, motor-evoked potential, corticospinal excitability, foot drop, plasticity

## Abstract

Corticospinal pathway and its function are essential in motor control and motor rehabilitation. Multiple sclerosis (MS) causes damage to the brain and descending connections, and often diminishes corticospinal function. In people with MS, neural plasticity is available, although it does not necessarily remain stable over the course of disease progress. Thus, inducing plasticity to the corticospinal pathway so as to improve its function may lead to motor control improvements, which impact one's mobility, health, and wellness. In order to harness plasticity in people with MS, over the past two decades, non-invasive brain stimulation techniques have been examined for addressing common symptoms, such as cognitive deficits, fatigue, and spasticity. While these methods appear promising, when it comes to motor rehabilitation, just inducing plasticity or having a capacity for it does not guarantee generation of better motor functions. Targeting plasticity to a key pathway, such as the corticospinal pathway, could change what limits one's motor control and improve function. One of such neural training methods is operant conditioning of the motor-evoked potential that aims to train the behavior of the corticospinal-motoneuron pathway. Through up-conditioning training, the person learns to produce the rewarded neuronal behavior/state of increased corticospinal excitability, and through iterative training, the rewarded behavior/state becomes one's habitual, daily motor behavior. This minireview introduces operant conditioning approach for people with MS. Guiding beneficial CNS plasticity on top of continuous disease progress may help to prolong the duration of maintained motor function and quality of life in people living with MS.

## Introduction

Over the past 15 years, the awareness of the importance of physical rehabilitation and exercise has been steadily growing in the field of multiple sclerosis (MS)-related research (1–3). This trend should continue, with ongoing development and testing of disease-modifying drugs (4), which will lead to prolonging disease stability and creating greater opportunities for reducing motor impairments, improving mobility, and improving quality of life in people with MS, as pointed out by Ploughman (3). Although underlying mechanisms may not be fully understood (5), mounting evidence indicates positive effects of exercise on physical fitness, balance and mobility, cognitive function, participation, and other outcomes (1, 6). A challenge is that a person with MS may not be able to appreciate the greatness of exercise, when reduced movement efficiency and impaired mobility make it difficult for him/her to be engaged in physical activity. Without changing what is available to execute essential daily motor function such as gait, and without changing what is limiting one's function, movement dysfunction would continue to limit mobility and quality of life in people with MS. While disease progress continuously alters one's physiology, it is essential to guide the central nervous system (CNS) plasticity that can help to prolong the duration of maintained motor function and quality of life in people living with MS.

In this brief review, we will discuss the corticospinal plasticity in people with MS and introduce operant conditioning approach as a method to target plasticity in the corticospinal pathway for improving motor function in people with MS.

## CNS Plasticity in People with MS

MS is a chronic inflammatory, autoimmune disease of the CNS. In persons with MS, neurological deficits are commonly attributed to inflammatory demyelination in the CNS and damage to the gray matter in cortical and subcortical structures, with lesion patterns, locations, volumes, and their rates of changes differing among subtypes of MS (7–10). In addition to accumulating structural damage, the process of inflammation itself affects synaptic transmission and plasticity (11). Elevation in the level of inflammatory cytokines not only changes glutamatergic and GABAergic transmissions, which lead to synaptic hyperexcitability and excitotoxicity, but also affects synaptic plasticity (11–14), which is essential for clinical and functional recovery. Thus, from damage to the brain and the descending pathways and from alteration in synaptic plasticity, disruption of corticospinal function is a hard-to-avoid problem in people with MS (15–17).

Transcranial magnetic stimulation (TMS), its motor-evoked potential (MEP), and their associated measures, such as short- and long-interval cortical inhibition (SICI and LICI), short-interval cortical facilitation (SICF), and intracortical facilitation (ICF), are useful tools for investigating cortical and corticospinal plasticity (18–22). They are also useful in detecting and predicting the progression of disability and recovery (15, 16, 23–27). For instance, small MEPs with long latencies, high motor thresholds, and prolonged cortical silent periods tend to correlate with the Expanded Disability Status Scale (EDSS) scores (15–17, 28–30). Silent period (SP) after MEP, known to reflect cortical inhibition at least partly (31–37), is reduced in the relapsing or progressive phases of MS (38, 39), whereas the SP is prolonged in the remitting phase (38). In the stable phase of relapsing–remitting MS individuals, SICI and ICF could be similar to those of the control group (17). A common observation is that cortical inhibition is reduced during the relapsing or progressive phase, whereas the inhibition is clearly present during the stable or remitting phase (11); the phase or state of disease appears to be reflected in the measured cortical inhibition. In addition, how these measures respond to plasticity-inducing neuromodulation can suggest the availability of plasticity at the time of assessment and help to predict recovery from relapse (11–13, 23, 40).

The availability of synaptic plasticity, also known as “plasticity reserve” (11), can be measured in persons with MS by applying plasticity-inducing neuromodulation techniques, such as repetitive TMS (rTMS) at high (e.g., 20 Hz) or low (e.g., 1 Hz) frequency, rTMS with intermittent or continuous theta burst stimulation patterns (iTBS and cTBS) (11, 13, 14, 41, 42), paired associative stimulation (PAS) with TMS, and peripheral nerve stimulation (PNS) (12, 23, 43, 44). They can be used to assess long-term potentiation (with high-frequency rTMS and iTBS), long-term depression (with low-frequency rTMS and cTBS), and spike-timing-dependent Hebbian-type plasticity (with PAS) (11, 13, 42). For example, in individuals with primary progressive MS, neither iTBS nor cTBS exert the expected plasticity effects; in individuals in the relapsing phase of MS, iTBS produces expected LTP effects, but cTBS fails to produce expected LTD effects (42). This plasticity reserve may be an essential mechanism of clinical symptom and disability progression in MS; when plasticity reserve is exhausted and synaptic plasticity is unavailable, surviving neurons would not be able to compensate for neuronal loss (11).

Importantly, while people with MS can display plasticity (43, 45–48), there is no guarantee that their plasticity adaptive to progressive neuronal damage is beneficial; it may exaggerate or lessen clinical symptoms (42). Thus, to guide the plasticity in beneficial directions, a neurobehavioral training should be incorporated into MS rehabilitation. For improving impaired motor function in people with MS, it would be critically important to induce and maintain beneficial plasticity in the corticospinal pathway, as its function is the foundation of voluntary and involuntary motor behaviors.

## Neuromodulation for Rehabilitation in People with MS

There are a wide variety of neurorehabilitation interventions currently available or being tested for individuals with CNS disorders, including MS (https://clinicaltrials.gov). Many of those expect to induce cortical and/or subcortical plasticity and may improve sensorimotor function [e.g., (49–52)]. Of different neuromodulation approaches, there have been growing interests in non-invasive brain stimulation (NIBS); in particular, rTMS and transcranial direct current stimulation (tDCS) have been increasingly utilized for treating various MS symptoms (41, 53–59). Other neuromodulation methods, such as deep brain stimulation and spinal cord stimulation, have been reviewed in (60). As effects and mechanisms of rTMS and tDCS have been thoroughly covered in recent reviews (54–56, 58), these methods will not be further discussed in this minireview. However, it is worth reiterating that studies of LTP or LTD-inducing rTMS (e.g., iTBS, low-frequency rTMS) and tDCS that affects polarization of the stimulated cortical network have shown some promising results; common MS symptoms, such as fatigue, cognitive functions, pain, and spasticity, can be alleviated by these methods (40, 41, 53, 54, 56–58, 61, 62).

When applying NIBS for improving impaired motor function, consideration on how to guide the stimulation-induced plasticity is critically important. Because NIBS-induced plasticity is rather widespread and not pathway specific, without an additional strategy to shape such plasticity into functionally beneficial changes, many changes at many different sites could compensate for each other, toward maintaining the state of neural network at net change of zero [i.e., homeostatic plasticity (63–69)]. Thus, pairing two interventions, e.g., iTBS + exercise (70), could be a logical NIBS application strategy for motor rehabilitation. Task-specific PNS, such as FES for foot drop (71–73), with which functional movement and phase-specific PNS occur concurrently, uniquely emulates a neuromodulation combination strategy, increases MEP amplitude, and improves motor function in people with MS and other neuromuscular disorders (49, 74).

Another class of neuromodulation methods include PAS (23, 43, 75–79) and operant conditioning of muscle [electromyographic (EMG)]-evoked potentials (80–82), which target plasticity in a specific pathway. Detailed mechanisms of PAS approaches have been discussed in (75, 76, 78, 83, 84). Briefly, with PAS protocols that induces spike-timing-dependent plasticity (76–78), synaptic transmission can be potentiated or depressed depending on the relative timing between the presynaptic and postsynaptic spiking (77, 85, 86), and repeated application of TMS-PNS PAS can potentiate corticospinal-motoneuronal transmission and excitability in people with MS (43, 45). A similar PAS concept can also be applied to cortical neurons (79, 87, 88). Therapeutic potency of PAS in people with MS is yet to be determined.

## Operant Conditioning of EMG-Evoked Potentials

Operant conditioning is a method for modifying a behavior based on the consequence of that behavior (89). Usually, when a person acquires a new behavior through operant conditioning, s/he does not need to discover the operant contingency through trial and error. However, when this approach is applied to a behavior of a neural pathway (e.g., a reflex), an individual must go through a trial-and-error discovery phase, as s/he would not have prior knowledge on how to control volitionally a behavior or the excitability of that specific pathway. Thus, with operant conditioning of an EMG-evoked potential that reflects the behavior and/or excitability of a certain neural pathway, a subject learns to produce a neuronal behavior that is rewarded through trial and error, similarly between humans and animals (89). Through repetition, the rewarded behavior can become a habitual behavior (90). With operant conditioning of an EMG-evoked potential, such as a reflex and an MEP, a subject is rewarded only for increasing or decreasing a target pathway's excitability (81, 82). Thus, over time, it changes the pathway that produces that response (82).

By changing the transmission of a key pathway with a directional aim (up/down), operant conditioning of an EMG-evoked potential seeks to improve the targeted pathway's function and enable more effective movements in which the targeted pathway contributes (91, 92). An emerging theory is that changing a key pathway leads to a cascade of wider beneficial changes in the activity of other spinal and supraspinal pathways (81, 93), impacting motor function recovery.

Much of the physiological and theoretical knowledge of operant conditioning approach is based on a large number of reflex conditioning studies (68, 82, 94). The most essential includes the following. An operantly conditioned reflex behavior rests on a hierarchy of plasticity from the brain to the spinal cord (68, 82, 95, 96). The reward contingency produces plasticity in the brain that induces and maintains the spinal cord plasticity that is directly responsible for the conditioned reflex behavior (68, 82, 94). Among the major descending pathways, the corticospinal tract is the only pathway essential for conditioning-induced plasticity (97). Thus, when the corticospinal tract and its plasticity are preserved at least partially, the targeted change can be induced through conditioning (98), which then changes how that reflex pathway functions in complex motion such as locomotion (80, 92, 99). These provide the foundation for currently emerging clinical applications of MEP operant conditioning.

## Operant Conditioning of the Motor-Evoked Potentials

**Figure 1:**
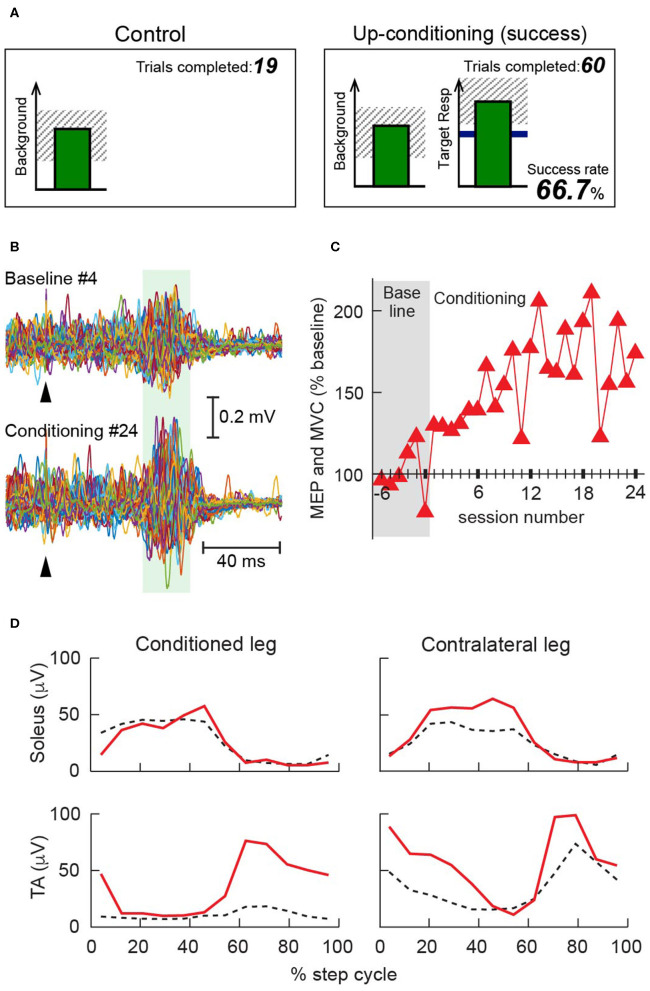
Operant conditioning of the tibialis anterior (TA) motor-evoked potential (MEP) in individuals with multiple sclerosis (MS) [modified from (104)]. **(A)** Visual feedback screens for MEP control and MEP operant conditioning trials. In all trials, the number of the current trial within its block is displayed, and the background electromyographic (EMG) panel shows the correct range (shaded) and the current value (green vertical bar, updated every 200 ms). If TA EMG activity stays in the correct range for at least 2 s and at least 5.5 s has passed since the last trial, an MEP is elicited. In control trials (left), the MEP panel is not shown. In conditioning trials (right), the shading in the MEP panel indicates the rewarded MEP range for up-conditioning. The dark horizontal line is the average MEP size of the baseline sessions, and the vertical bar is the MEP size, calculated in the MEP interval of that specific individual [e.g., 45–70 ms after transcranial magnetic stimulation (TMS)], for the most recent trial. The vertical bar appears 200 ms after TMS. If that MEP size reaches into the shaded area, the bar is green, and the trial is a success. If it falls below the shaded area, the bar is red, and the trial is a not a success. The running success rate for the current block is shown at the bottom. **(B)** Examples of TA MEP in a 56-year-old woman with MS (Expanded Disability Status Scale 4.0* at baseline). Peristimulus EMG sweeps from the fourth baseline session (top) and the 24th conditioning session (bottom). For each part, 75 sweeps are superimposed. A green shaded band indicates the time window for her MEP size calculation. Arrowheads indicate the time of TMS. **(C)** Mean MEP size (i.e., the mean of 225 control MEP trials in baseline sessions or 225 conditioned MEP trials in conditioning sessions) in 6 baseline and 24 conditioning sessions that occurred at a rate of 3 sessions/week. Over the course of conditioning, her MEP size increased progressively; the final MEP size was 175% of the baseline value. **(D)** Rectified locomotor EMG activity in soleus and TA bilaterally before (dashed black) and after (solid red) conditioning. The step cycle, from foot contact to the end of swing phase, is divided into 12 equal bins. After TA MEP up-conditioning, swing phase TA burst increased in the conditioned leg, which helped this individual regain ankle dorsiflexion and eliminated foot drop. The swing phase burst was also increased in the contralateral TA. All panels have been adapted from (104) with permission. *EDSS 4.0 (105): Fully ambulatory without aid, self-sufficient, up and about some 12 h a day despite relatively severe disability consisting of one FS grade 4 (others 0 or 1), or combination of lesser grades exceeding limits of previous steps; able to walk without aid or rest some 500 m.

As in reflex operant conditioning (68, 81, 82, 100–102), operantly up-conditioning the MEP can increase the corticospinal excitability for the targeted muscle in people (91, 103). In the first 100–1,000 up-conditioning trials, a person learns through trial and error how to increase MEP size, and MEP size gradually increases over the subsequent conditioning sessions (Figure 1). Motivation is critical in operant conditioning (89, 90, 106, 107); the person must value the positive feedback that s/he receives from producing a larger MEP. Our studies suggest that in individuals with CNS disorders, who take the conditioning trials more seriously than those without CNS injury, MEP size is highly likely to increase, and their MEP increase can persist at least a few months after conditioning ends (103, 104).

Two key factors underline the therapeutic potency of MEP conditioning. First, it targets plasticity to the corticospinal pathway that produces an MEP in the targeted muscle. The protocol prohibits change in the background EMG activity; the individual is rewarded only for increasing the target muscle's MEP (i.e., for increasing corticospinal excitability for the target muscle). This pathway specificity differentiates MEP conditioning from EMG biofeedback training (108–112) or muscle strength training (113–119), both of which are not tailored for modulating or controlling the excitability or behavior a specific pathway. While practice is essential in improving motor performance, movement practice alone could let an individual easily default to relying on what is readily available (e.g., trying to rely on the hip flexors, instead of improving corticospinal drive to the impaired ankle dorsiflexors), leaving a key pathway unchanged. Because MEP up-conditioning increases the excitability of the corticospinal pathway for the target muscle, it affects motor skills, such as locomotion, to which the pathway contributes. Thus, with the ankle dorsiflexor tibialis anterior (TA) MEP up-conditioning (Figure 1), locomotion can be improved in people suffering from foot drop (weak ankle dorsiflexion) (91, 104).

Second, by improving the function of a key pathway, corticospinal pathway, MEP conditioning can trigger further beneficial changes in the activity of other CNS pathways (80, 81, 93, 120), changing what is possible/available in one's recovery path. By targeting the weakened corticospinal drive to the TA and ameliorating the locomotor impediment of foot drop, TA MEP conditioning can enable more effective execution of locomotion; this would then induce wider beneficial plasticity. Increased corticospinal drive to the conditioned TA (49) can explain increases in TA MEP and TA burst amplitude during the swing phase of locomotion observed in people with MS and SCI (91, 104) but cannot explain widespread bilateral improvements in locomotor EMG activity (91, 104) (Figure 1). These wider effects of MEP conditioning are similar to those of the soleus H-reflex down-conditioning, with which proximal and distal leg muscles' locomotor EMG improved bilaterally in people with SCI (92). How an operant-conditioning acquired new skill of changing a specific pathway's excitability would trigger a widespread adaptive plasticity in many spinal/supraspinal pathways has been addressed in a theory of system function known as the negotiated equilibrium model (68, 93).

## Effects of MEP Conditioning and Corticospinal Plasticity in People With MS

Among people with foot drop due to MS or SCI, locomotor TA activity improved and walking speed increased while MEP increased (91, 103, 104). Conditioning-induced MEP increase was often accompanied by systematic decrease in SP duration (103, 104). SP is known to reflect cortical inhibition at least partly (31–37), and different neural circuits underlie MEP and SP (121–123). If MEP up-conditioning simply increased the general excitability of the cortex, both MEP and SP would have increased [e.g., (124)]. This was not the case. Instead, there were some selective effects on excitatory and inhibitory neurons in the cortex (125, 126). Since reduction in intracortical inhibition occurs through modulation of GABAergic inhibitory interneurons (127–131), it is highly likely that GABAergic inhibitory mechanisms are involved in conditioning-induced SP changes. SPs are often prolonged in people with stable or secondary progressive MS (28, 29, 132), which likely reflects altered GABA_B_-mediated intracortical inhibition (33, 131, 133, 134). Despite an altered state of cortical inhibition in preconditioning, MEP up-conditioning could reduce SP in individuals with stable MS (104). Further investigation is clearly needed to understand the mechanisms and effects of MEP up-conditioning on cortical inhibition in MS.

## Operant Conditioning of Spinal Reflexes

In addition to MEP conditioning protocols, several reflex conditioning protocols are currently being developed. To date, two protocols have been systematically tested in people with or without CNS damage: the soleus short-latency stretch reflex (known as M1 response) conditioning, using mechanical joint perturbation (135), and the soleus H-reflex conditioning, which uses electrical stimulation of the tibial nerve (92, 99, 102, 136). With both stretch and H-reflex conditioning protocols, the person learns to increase or decrease the target reflex size over 24–30 conditioning sessions. The protocols are designed to induce sustaining changes in descending influence over the reflex pathway, which in turn, produce targeted plasticity in that pathway (101). Because these protocols can change the transmission of targeted pathways, they can be designed to address the specific functional deficits of an individual. For example, in people with spastic hyperreflexia due to incomplete SCI, down-conditioning of the soleus H-reflex pathway, whose hyperactivity impaired locomotion, could improve their locomotion (80, 92). Down-conditioning of the stretch or H-reflex might also improve spasticity and spastic movement disorders in people with MS (137). It should also be possible to condition other important pathways, such as pathways of spinal reciprocal and presynaptic inhibition (138–140), for further improving their motor functions.

## Operant Conditioning in MS: Challenges and Possibilities

Up until now, the majority of evoked potential operant conditioning studies have been done in SCI (68, 80, 82, 92, 99, 141–145), and its investigation in MS is still in an early stage. Unique challenges in the MS population that do not necessarily apply to the SCI population include impaired cognitive function, fatigue, and ongoing and/or recurring inflammation (14, 56, 59, 146). Since operant conditioning is a behavioral learning approach (81, 82, 89), impairments in learning, memory, and attention that are frequently found in MS may affect the effectiveness of this approach in people with MS. The fact that recurring inflammation influences synaptic plasticity and plasticity reserve (11, 13), which are physiological mechanism of learning, memory, and function recovery, could well interfere with induction and maintenance of conditioning-induced beneficial plasticity. Furthermore, extents of these challenges could vary among MS subtypes and across different individuals (11, 17, 38, 42). Clearly, more studies are needed to determine the applicability of operant conditioning approach in people with MS, and an investigation needs to include persons with all MS subtypes. Long-term follow-up should also be part of such investigations, although often unpredictable disease progress may mask or reduce the induction of plasticity and function improvements temporally or permanently (23, 44, 49, 74). Over 3.5 years of follow-up with a woman with secondary progressive MS supports a possibility of long-term maintenance of corticospinal transmission and function improvements with MEP operant conditioning (104).

A possible strategy to overcome the above-mentioned MS-related challenges is coadministration of conditioning training with NIBS or pharmacological treatment. Reflex or MEP conditioning that aims to change behaviors of the targeted pathway is fundamentally different from rTMS and tDCS, or pharmacological treatments, such as baclofen (147, 148). Because the mechanisms of action differ so vastly from each other, with careful consideration of dosing schedules and individual or combined effects, it may be possible to enhance functional outcomes by coadministering a conditioning protocol with another intervention. Drugs such as dalfampridine and d-aspartate (149–153) may further enhance the corticospinal plasticity and transmission improvement produced by MEP conditioning.

## Conclusion

A growing number of neurophysiological studies indicate the importance of neuroplasticity and its management for neurorehabilitation in people with MS (11, 13, 54, 56–60, 154). While the benefit of exercise in health and wellness has become recognized (1, 6), investigation on how to improve impaired motor function and mobility, which can limit one's ability to exercise, has been left behind (3). Applying neural training methods, such as operant conditioning of EMG-evoked potentials, to guide beneficial plasticity in the corticospinal or other important CNS pathways may minimize the factors that limit function improvement in people with MS. As CNS plasticity remains available over many years of disease progress (43, 46, 47), guiding it appropriately to gain function improvements on top of changing physiology may help to prolong the duration of maintained motor function and quality of life in people with MS.

## Author Contributions

AT drafted, edited, and revised the manuscript. TS edited and revised the manuscript. Both authors read and approved the final version submitted for publication.

## Conflict of Interest

AT holds several patents related to operant conditioning of spinal reflexes (US patent number 8862236, 9138579, and 9545515). These patents are not providing income in any form. The remaining author declares that the research was conducted in the absence of any commercial or financial relationships that could be construed as a potential conflict of interest.
